# Announcment: 31st International Conference on Coordination Chemistry

**DOI:** 10.1155/MBD.1995.339

**Published:** 1995


					31ST INTERNATIONAL CONFERENCE ON
COORDINATION CHEMISTRY
The University of British Columbia, Vancouver, Canada
August 18-23, 1996
ORGANIZING COMMITTEE
Chris Orvig, University of British Columbia, Chairman
Ross Hill, Simon Fraser University, Secretary
Rowena Tate, UBC Conference Centre, Conference Manager
PROGRAMME COMMITTEE
William R. Cullen, University of British Columbia
Michael D. Fryzuk, University of British Columbia
Ross Hill, Simon Fraser University
Brian R. James, University of British Columbia
A.D. Kirk, University of Victoria
Peter Legzdins, University of British Columbia
Alex McAuley, University of Victoria
Chris Orvig, University of British Columbia
Derek Sutton, Simon Fraser University
SCIENTIFIC PROGRAMME
Plenary Lecturers
Prof. C. David Garner, University of Manchester, UK; "X-ray Absorption Spectroscopic Studies of
Metal Centres in Biology and Developments of Relevant Synthetic Analogues"
Prof. Brian R. James, University ofBritish Columbia, Canada; "Coordination Chemistry, and Catalytic
Conversions, of Hydrogen Sulfide"
Prof. Eiichi Kimura, Hiroshima University School ofMedicine, Japan; "Macrocyclic Metal Complexes
for Selective Recognition of Nucleic Acid Bases and Manipulation of Gene Expression"
Prof. Walter G. Klemperer, University ofIllinois, USA; "Self-Assembled Inorganic Monolayers: Co-
ordination Chemistry in Two Dimensions"
Prof. Leonard F. Lindoy, James Cook University, Australia; "Tailoring Macrocycles and Molecular
Assemblies for Metal Ion Binding"
Prof. Richard R. Schrock, Massachusetts Institute ofTechnology, USA; "High Oxidation State Coordi-
nation Chemistry with Triamidoamine Tungsten and Molybdenum Complexes"
339
Session Lecturers
There will be nine sessions covering all aspects ofcoordination chemistry; session lecturers are as follows
(as of the time of printing):
1. Metal Centres in Biology and Medicine
Dr. Michael Abrams, Johnson-Matthey, Inc., USA; "Ruthenium Complex Scavenging ofNitric Oxide in
Biological Systems"
Prof. Peter J. Sadler, Birkbeck College, University of London, UK; "Metallodrugs: Design and Mecha-
nism ofAction"
Prof. Dr. Dieter Sellman, Universit/it Erlangen-Ntirnberg, Germany; "Biological Challenges to Coordina-
tion Chemistry: Modelling the Reactivity of Nitrogenase and Other Metal Sulfur Enzymes"
Prof. Edward I. Solomon, Stanford University, USA; "Electronic Structure of the Blue Copper Active
Site: Contributions to Electron Transfer Reactivity"
2. Mechanistic Insights
Prof. James H. Espenson, Iowa State University, USA; "Mechanisms of Reactions Catalyzed by
Methylrhenium Trioxide"
Prof. Hitoshi Ohtaki, Ritsumeikan University, Japan; "Structure of Intermediates in the Metal Substitu-
tion Reactions of the Mercury(lI)-Porphyrin Complex with Divalent Transition Metals Determined by the
StoppedFlow-EXAFS Method"
Dr. Joop A. Peters, Delft University ofTechnology, The Netherlands; "Solution Structures ofLanthanide
Chelates"
Prof. Alexander D. Ryabov, M. V. Lomonosov Moscow State University, Russia; "Mechanisms of
Enzymatic Reactions of Ferrocene Derivatives"
Prof. Thomas W. Swaddle, University of Calgary, Canada; "Understanding Pressure Effects in the Solu-
tion Chemistry of Coordination Compounds"
3. Activation ofSmall Molecules
Prof. Pierre Braunstein, Universit6 Louis Pasteur, France; "Bimetallic Synthesis and Reactivity"
Prof. Ernesto Carmona, Universidad de Sevilla- CSIC, Spain; "Rhodium and Iridium Mediated C-C
Bond Forming Reactions Involving Small, Unsaturated Organic Molecules"
Prof. Juan Costamagna, University of Santiago, Chile; "Complexes with Aza-macrocyclic Ligands Ac-
tive for the Reduction of Carbon Dioxide"
Prof. William B. Tolman, University of Minnesota, USA; "Binding and Activation of Dioxygen and
Nitrogen Oxides by Copper Complexes" Relevance to Biological Catalysis"
Prof. Joan S. Valentine, University of California at Los Angeles, USA; "Defining Possible Mechanisms
for Enzymatic Dioxygen Activation by Studying Reactions of Transition Metal Complexes"
4. New Materials
Prof. Mark P. Andrews, McGill University, Canada; "Coordinating Molecules and Mesoscopic Struc-
tures for Optical Devices"
Prof. Michael J. Sailor, University ofCalifornia at San Diego, USA; "Chemistry at the Surface of Lumi-
nescent Porous Silicon"
Dr. Lynn Schneemeyer, AT & T Bell Laboratories, USA "Chemistry of Solids"
Prof. Samuel I. Stupp, University ofIllinois, USA: "Self-Assembly ofMolecular and Composite Materi-
als
340
5. Multidentate Ligands and SupramolecularAssemblies
Dr. Paul Beer, University of Oxford, UK; "Cation and Anion Coordination Chemistry of Redox- and
Photoactive Ligand Systems"
Prof. Makota Fujita, Chiba University, Japan; "SelfAssembling Supramolecular Complexes: From
Macrocycles to Catenanes"
Prof. Stephen Lincoln, University ofAdelaide,Australia; "PendantArm Macrocyclic Ligand Complexes"
Prof. Vincent L. Peeoraro, University of Michigan, USA; "Metallacrowns: New Inorganic Molecular
Recognition Agents"
6. Electronic Properties: Theory and Practice
Prof. Odile Eisenstein, Universit6 de Paris-Sud, France; "AnAb Initio Calculation ofthe NMR 1H Quan-
tum Exchange in the Case of Osmium Hydride Complexes"
Prof. Olivier Khan, Institut Universitaire de France; "Molecular Magnetism: a New Challenge for Coor-
dination Chemists"
Prof. A. B. P. Lever, York University, Canada: "Electrochemical Parameterization: Theory, and Applica-
tion to Organometallic Species"
Prof. Per E. M. Siegbahn, University of Stockholm, Sweden; "Theoretical Transition Metal Chemistry"
Methods, Models and Results"
7. Environmental Chemistry: Initiatives andApplications
Dr. O. F. X. Donnard, Universit6 de Bordeaux 1, France; "EnvironmentalAspects ofthe Biogeochemistry
of Sn, Se and As in Marine Ecosystems"
Dr. Donald S. Galnble, Agriculture Canada; "Discrete Site Complexes On Humic Polyelectrolytes: The
Frontier of Coordination Chemistry"
Prof. Stalfan Sjoberg, University of Umea, Sweden; "Surface Complexation Models for Sorption at the
Mineral Water Interface"
Prof. Dr. Rudy van Eldik, Universitit ErlangenNurnberg, Germany; "Metal-catalyzed Oxidation ofS(IV)
A Challenge to Coordination Chemists"
8. Main Group Chemistry
Prof. Andrew R. Barron, Rice University, USA; "Aluminum, Gallium and Indium Complexes of Group
16 Donor Ligands"
Prof. Phillip P. Power, University of California at Davis, USA; "Multiple Bonding and Steric Effects in
the Main Group 3 Elements"
Prof. Dr. Hubert Sehlnidbaur, Technische Universitit Munchen, Germany; "Metalloids in Multinuclear
Metallo Complexes"
Prof. James D. Wuest, Universit6 de Montr6al, Canada; "Coordination Chemistry of Multidentate Lewis
Acids"
9. Advances in Molecular Structure and Design
Prof. C. C. (Kit) Cummins, Massachusetts Institute of Technology, USA; "Reactions of Three-Coordi-
nate Transition Metal Complexes with Small Molecules and Related Substrates"
Prof. Kiln R. Dunbar, Michigan State University, USA; "Ordered Arrays of Metals With Polynitrile or
Cyanide Ligands
Prof. Dr. E Ekkehardt Hahn, Freie Universitat Berlin, Germany; "Ligands with Subvalent Group 14
Donor Atoms: Multidentate Isocyanides, Carbenes, and Stannylenes"
Dr. Maurizio Peruzzini, ISSECC CNR, Italy; "Synthesis of Aminophosphine Ligands and Their
Organometallic Chemistry in Combination with Transition Metals"
341
Poster Sessions and Short Lectures
Poster sessions and 20-minute lectures will be culled from the submitted abstracts. These will also be
divided into the nine session topics listed above.
More information can be obtained from the Conference Secretariat
31st ICCC Secretariat
UBC Conference Centre
5961 Student Union Boulevard
Vancouver, B.C. Canada V6T 2C9
Telephone" 1 (604) 822-1050
Facsimile: 1 (604) 822-1069
E-mail" registration @ brock.housing.ubc.ca
342

				

